# DrawingOut – An innovative drawing workshop method to support the generation and dissemination of research findings

**DOI:** 10.1371/journal.pone.0203197

**Published:** 2018-09-20

**Authors:** Sofia Gameiro, Berit Bliesemann de Guevara, Elisabeth El Refaie, Alida Payson

**Affiliations:** 1 School of Psychology, Cardiff University, Cardiff, United Kingdom; 2 Department of International Politics, Aberystwyth University, Aberystwyth, United Kingdom; 3 School of English, Communication and Philosophy, Cardiff University, Cardiff, United Kingdom; 4 School of Journalism, Media and Culture Studies, Cardiff University, Cardiff, United Kingdom; University of Leicester, UNITED KINGDOM

## Abstract

A growing body of literature has explored the potential for arts-based methods to generate and disseminate research, particularly on sensitive or complex topics. This article presents DrawingOut, a metaphor-centred drawing workshop designed to collect visual and textual data about individuals’ experiences of sensitive or taboo health experiences. The visual data, consisting of the artwork produced by participants, and the textual data, all conversations and discussions occurring during the workshop, can also be used to create engaging outputs for dissemination. We piloted DrawingOut in a study of nine women with a minority ethnic or religious background in Cardiff, UK. The women were invited to participate in a series of structured drawing activities. The conversations occurring during the workshop were recorded and then subjected to thematic analysis. Results showed that women’s views and experiences could be grouped in eight major themes covering their wellbeing, relationship with others, and healthcare views and experiences. We produced an A5 16-page booklet that presents the pilot study results, illustrated with participants’ own drawings and quotes, which was disseminated among participants, their community and other relevant stakeholders. This pilot study supports the view that healthcare actors can use the DrawingOut method to engage people to talk about sensitive health topics, while simultaneously providing them with an enjoyable and empowering research experience. In our pilot study the DrawingOut was successful in eliciting rich visual and textual data that captures a diversity of views and experiences, with the added benefit that these can be used to produce engaging outputs for dissemination.

## Introduction

The ability to provide appropriate and equitable healthcare requires practitioners to have insight into the personal meanings patients attribute to their health condition. The view adopted here is that disease and health experiences are framed by social, cultural, economic, political and environmental factors [1], and that these factors can only be fully understood by adopting a constructivist approach that seeks to capture how individuals within a particular context perceive and create meaning of their experiences of health/disease [2]. Qualitative techniques, such as interviews and focus groups, have been increasingly used in health research to capture such psycho-cultural-social determinants of health and care experiences [3]. Although widely used, these techniques have limitations. First, they are highly dependent on linguistic proficiency and verbal fluency, which means that they need to be adapted when working with participants who, for whatever reasons, lack these skills. Second, the use of explicit questions inherent in these methods may frame the discussions in a way that restricts participants’ ability to set their own agendas. Finally, some topics involve such complex experiences and emotions, and/or are governed by such strong cultural taboos and shame, that they may resist objectification in language.

In this article, we present DrawingOut—an innovative metaphor-centred drawing method to support the generation and dissemination of research findings. The innovative aspects of this method consist in the use of drawing as a central tool for data collection; the use of visual metaphors as the main knowledge production stimulus presented to participants (instead of questions in interviews or focus-groups); and the compilation of the visual and textual data generated into a visually-appealing output used to disseminate findings. We start by discussing the advantages and limitations of drawing as a research tool, before presenting the DrawingOut method and illustrate its use in a pilot-study that investigated the infertility experiences of nine women with a minority ethnic or religious status living in Cardiff, UK.

### Drawing as a research method

Drawings have been used in health for many decades to enhance verbal educational messages or as a therapeutic technique. Only relatively recently, however, have drawings been used as a research tool, with many arguments put forward to justify its use.

From an equality and diversity perspective, drawings are an important way of overcoming linguistic barriers in communication. They may thus facilitate the engagement in research of marginalized or vulnerable groups with lower levels of proficiency in the language of care, or in multilingual contexts. For instance, McKillop and colleagues [4] used visual approaches to work collaboratively with immigrant workers to develop a poster with health and safety messages. Participants considered the combination of drawings and minimal text easier to understand than text-only posters.

The request to draw offers participants freedom in choosing their own perspectives and agendas. However, some people may find drawing challenging and intimidating, and this may have the unintended effect of increasing participants’ resistance to engage with research. In a study where women were invited to draw images related to their experience of chronic vaginal thrush, for instance, a number of participants apologized for their poor drawing skills and expressed a reluctance to engage with the activity [5]. This may be particularly problematic when the visual depiction of a topic is technically difficult or embarrassing, for example, because it involves drawing ‘private’ body parts. However other studies show that, when the participants do engage in drawing, this may facilitate the process of knowledge creation. Guillemin [6] argues that, in the act of drawing, participants simultaneously construct knowledge about the content or topic of the drawing. Work in experimental psychology supports this view, with studies showing that children up to the age of 15 who are asked to draw and tell about an emotional event reveal more than twice as much verbal information than children who are asked to just talk about the event [7, 8].

In this context, eliciting visual-metaphors, defined as visual representations of concrete entities to draw attention to their non-literal nature [9], may further facilitate knowledge creation. Indeed, multiple research shows that patients naturally use metaphors to convey their health/disease experiences, for example, of cancer [10, 11] and depression [12, 13]. The specific metaphors people choose are shaped by their cultural backgrounds and social contexts, as well as their own values and unique experiences [14]. However, some of the most common verbal metaphorical expressions relating to the illness experience (e.g., fighting a war) have become so conventionalised over time that they are difficult to avoid, even if they may not, in fact, adequately reflect the experiences of individual patients. We thus hypothesized that, when people are able to express their views and emotions about complex health issues through visual metaphor, they are less constrained by linguistic conventions and thus perhaps better able to express meanings that may be outside of or alternative to dominant cultural meanings, and to convey their own unique meaning-making practices [15].

It is also important to note that methods based on verbal conversations may hamper the communication of emotion-laden experiences that were not coded verbally when originally stored in memory [16]. Drawing can facilitate the retrieval process of such emotionally charged memories. Indeed, drawing as a projective technique was originally developed by psychologists for patients who either resist revealing or who are not aware of their underlying motives, urges and intentions [17]. Therefore, drawing can be a useful tool to help people express their views about sensitive or taboo topics.

Finally, drawings have immense potential for knowledge dissemination. They are able to communicate different levels and types of individual experiences in an immediate, striking way [18], and to share complex research findings in an engaging and non-technical manner with people who are resistant towards or unfamiliar with scientific language. This is consistent with results from a systematic review of the role of pictures in improving communication, which showed that the use of drawings, especially when they are closely linked to related verbal messages, is associated with higher attention to and recall of health education materials, compared with text alone [14]. Drawings have also proved to be an effective tool for facilitating critical reflection and dialogue around sensitive health issues [15], for instance, sexual dysfunction in cancer [19].

In sum, using visual metaphors to elicit drawings as a data collection and dissemination tool can provide an effective means of exploring health topics, in particular (but not exclusively) if these are of a sensitive nature and when working with minority or vulnerable groups, because drawings are more inclusive than verbal-only methods, their projective, non-directive character promotes new knowledge creation and sharing, and they facilitate broader engagement with the research findings.

## The DrawingOut method

### Aims and procedures

The DrawingOut method consists of a one-day metaphor-centred drawing workshop to collect visual and textual data about a particular health related topic. The visual data is the artwork produced by participants and the textual data consists of all conversations and discussions occurring during the workshop. The collection and analysis of these two types of data have, beyond the typical research aim of producing knew knowledge about a particular topic, the express purpose of creating engaging outputs for dissemination purposes.

DrawingOut was designed to be used with minority or vulnerable patient groups or to explore sensitive health topics. However, in practice it can be used with any patient population, as long as they have basic drawing abilities. Indeed, it assumes that everyone can draw, and that simple drawings can convey very powerful messages. The workshop starts with two sessions with very simple exercises to introduce participants to the basics of drawing (things and people; and thoughts and feelings). In this way participants get acquainted with the medium, and possible concerns or resistance can be addressed before participants are asked to produce their own drawings. Despite its inclusive nature, it is important to consider the implications of working with groups for whom drawing skills may not be fully developed. DrawingOut users may consider the use of other visual art forms more suitable for their target participants (e.g., photography, water colours, collage, etc).

The DrawingOut method is structured around a series of activities that ensure a phased introduction to drawing and thereby facilitate participants’ active engagement. It then progresses to present metaphor-based exercises to engage participants in drawing their experiences and views:

1. Discussion of topic-related images. Participants are presented with a set of pre-selected images about the topic under analysis. The images need to express something (e.g., an experience, feeling, fact) about the topic and may be selected to encompass broad themes that the researchers want to focus on (e.g., symptoms, social responses). Participants are divided into groups of 3–5 people, and each participant is asked to choose the picture they most identify or connect with, describe it to the group and explain why they selected it. This is followed by a large group discussion, where each image is projected onto a screen and participants are asked to comment on it.
2. The basics of drawing: How to draw things and people. Participants are introduced to the basics of drawing objects and people in a PowerPoint-based lecture and guided through some simple drawing exercises. More specifically, they are given a sheet of paper with 12 circles and asked to transform the circles into, first, an object of their choice, second, a face expressing an emotion and, finally, their own self-portrait. They are also asked to use colours as appropriate (e.g., to express emotions). Then one of the participants or facilitators acts as a life model, striking a range of different poses for the others to draw, so that participants are introduced to simplified ways of drawing the human figure. Participants are then encouraged to use speech or thought balloons and words written in lettering that matches the thoughts and emotions expressed. This session ends with the request to ‘Draw yourself thinking or talking about the topic under analysis’ (self-portrait exercise).
3. The basics of drawing: How to draw thoughts and feelings. Participants are introduced to the concept of visual metaphor, defined simply as the use of something visible to show something that is invisible. Several examples of visual metaphor are presented and participants are prompted to discuss their potential meanings. Then participants are invited to represent the research topic through a range of visual metaphors and share with the group. Examples of questions are: ‘If your symptoms were a creature or animal, what would they be?’; ‘If your health condition was a place, what would it be?’, ‘If your symptoms were an object, what would it be?’. These questions should focus the participants on the topic under analysis, while giving them freedom to choose what particular aspect they want to represent, and how.
4. Free drawing session. Participants are given time to produce a large-scale drawing about any aspect of their illness experience.
5. Group sharing. The workshop ends with a group session in which participants are invited to present their artwork and comment on the different views emerging.

Although the workshop is carefully structured, the format also allows for spontaneous interactions and discussions at any time. It is therefore advisable to use several audio recorders to capture both the small- and large-group discussions. It may also be useful to take observational notes in order to support the transcription process of all conversations and discussions and the pairing of the art work with the explanations being presented.

The number of workshops run and participants included in each workshop will depend on the goals of the research, but we advise not to have more than 12 participants per workshop. This makes the implementation of the workshops feasible, while providing scope to collect a range of different viewpoints without participants having to compete for ‘air time’.

An important aspect to consider is the facilitation of the workshop. The facilitator(s) will have to lead the workshop. This involves introducing participants to the workshop method and aims, guiding them through the different activities, delivering the PowerPoint-based lectures, keeping the group on time and focused on the topic, encouraging participation from all attendants, and summarising discussions from time to time to check understanding of participants’ comments. Although no specific training is required, it is advisable that the facilitator(s) is/are familiar with the workshop materials and, more generally, with group-based methods of qualitative data enquiry.

The data collected with DrawingOut is of an audio/textual and visual nature. It is beyond of the scope of this paper to address all the analytical possibilities that the data offer. In brief, it is expected that the researchers’ epistemological position and research questions will ground their analytical work. In our view, DrawingOut is innovative precisely because it allows the pairing of the themes that emerge from the textual data with powerful and poignant images. However, researchers may opt to consider the visual data as a by-product of the method used, intended solely to facilitate the production of the textual data.

DrawingOut was designed to enable engaging dissemination of findings by using the participants’ artwork. How researchers/facilitators choose to use the artwork (and with what specific goals) is entirely up to them. For instance, researchers can create booklets, movies, posters, websites, blogs, etc.; they may choose to use each artwork produced independently or group them under common themes, and they may present the artwork in isolation or together with the textual data collected. We advise that researchers obtain feedback from the workshop participants before disseminating the artistic outputs to a larger audience in order to avoid misrepresentations.

### Copyright and ethical considerations

The use of drawings raises some ethical issues, including creative rights and permissions. DrawingOut users will need to look for a portfolio of drawings to use in activity 1, either via direct contact with artists or via research (internet, libraries, etc.); in both cases, the copyright of the drawings used will need to be considered. In general, researchers can use copyrighted artwork for non-commercial research without seeking permission from the author. However, it is the responsibility of the DrawingOut users to ensure they are complying with the law.

DrawingOut assumes that the artwork produced by the participants will be used for dissemination purposes. Therefore, informed consent for this specific aim will need to be obtained from participants. Another aspect to consider is ensuring the anonymity of participants (unless they are happy to be named). At the minimum, this implies that the artwork cannot be signed, but it is also important to examine the artwork to ensure that there are no identifying elements that can be linked back to participants.

## Pilot study

Around 10% of people see their parenthood desires compromised by a diagnosis of infertility [20]. For most people the inability to conceive represents a serious blow to their family building aspirations, triggering intense feelings of anxiety, loss and grief, and bringing into question their sense of self as individuals and partners. Infertile people tend to feel misunderstood in their grief and isolated from what they label ‘the fertile world’, which often includes members from their own family, close friends and community. Around half of all infertile people undergo medical treatment to conceive [20]. These patients find it very hard to cope with the highly technological and complex procedures that treatment entails. Patients have to interact and share intimate information with a large team of experts, and submit themselves to painful and physically intrusive diagnostic and treatment procedures that tend to extend over long periods of time. Treatment usually involves more than one attempt, and each attempt is costly but offers only around 30% chances of success. Overall, infertility and its treatment are thus a challenging experience for both patients and their partners, with around 20% suffering from anxiety or depression disorders and 22% giving up without achieving parenthood [21–23].

We welcomed the opportunity to research this sensitive topic by trialling the DrawingOut method with a heterogeneous group of women with a minority ethnic or religious status in the UK. We wanted to understand these women’s individual and relational experiences of infertility and related healthcare, and to share the findings with the women’s communities and relevant stakeholders in an engaging way.

We considered DrawingOut to be an appropriate method to use because of these women’s heterogeneous backgrounds and (in some cases) limited English proficiency. Evidence showing sociocultural variation in the metaphors used to describe infertility experiences also made the use of DrawingOut attractive. While in Western societies infertile patients are typically framed as winners or losers in a game, lottery, or race against the biological clock, patients themselves often prefer to conceptualize the process as a journey, battle, or a job involving the goal-directed investment of their bodily and monetary resources [24–26]. In other cultures, however, different metaphors have been observed; for instance, women in 1980s rural Cameroon expressed their reproductive health concerns through the use of violent imagery involving plundered kitchens and the interference by supernatural forces in the preparation of food [27]. We therefore anticipated that the DrawingOut method would allow us to capture common themes in the women’s views and experiences, while also documenting variations in the metaphors used to express these experiences.

### Participants

Participants were nine adult women with a minority ethnic or religious status in the UK, who were currently experiencing or had experienced infertility in the past. The average age was 42 (range 30–59). Five women were South Asian Muslims, two were Sub-Saharan African Christians, one was a North African Muslim, and one a British Muslim married to a North African man. Four women reported experiencing fertility problems in the present and five in the past. Most of the latter had managed to conceive but one was still childless, while another was trying to have a second child and again experiencing fertility problems. The participants had different levels of English proficiency, ranging from native- and near-native to very limited skills only.

### Procedures and analysis

We considered all copyright and ethical issues inherent in running the group workshop and to use participants’ artwork for dissemination purposes, and obtained ethical approval from the Ethics Committee of the School of Psychology, Cardiff University. All women were asked to sign a consent form and were debriefed at the end.

We adapted the general and flexible procedures of DrawingOut in order to address two research questions: ‘How does infertility affect the wellbeing of these women and their relationships with their partner, family and community?’; ‘What are these women’s views and experiences regarding fertility healthcare?’. The workshop started with an ice-breaking activity to allow participants and researchers to introduce themselves. Rules of good conduct were discussed to ensure consideration and confidentiality during and after the workshop. For the first activity, discussion of research topic-related images, we used a set of infertility-related comic strips by illustrator Paula Knight (available online [28]). For the third activity, the basics of drawing thoughts and feelings, the women were encouraged to draw infertility as a creature/animal, a place and weather conditions, respectively

To identify common views and experiences we conducted a thematic analysis of the textual data, following the procedures recommended by Braun and Clarke [29]. A bottom-up approach to the data was adopted, in which SG and BBG first familiarised themselves with the full workshop transcript; second, individually assigned textual descriptors to relevant passages in a line-by-line coding; and third, discussed the descriptors and grouped them into themes, which had to be closely linked to the data and capture a patterned response or meaning within it [29]. Finally, the themes were grouped into higher-order themes. To ensure reliability, SG and BBG first discussed the textual descriptors and themes until agreement was reached regarding their relevance and the appropriateness of their labels. After that, they repeated this process by reviewing the themes against the original data. Finally, they presented their coding to the other members of the research team (who had attended the full workshop) for a final review. At a later stage, the workshop participants were also asked to comment on whether they felt the identified themes accurately represented their views and experiences.

### Results

During the DrawingOut workshop there were four activities where women were prompted to share their views and experiences of infertility by either discussing images about infertility or by doing their own infertility related drawings. These activities were: 1) discussion of infertility related images, 2) self-portrait exercise; 3) visual metaphor drawing exercises; and 4) free drawing session (and group sharing). It was through the thematic analysis of the textual data collected during these activities that we identified themes reflecting commonalities in these women’s views and experiences of infertility. It is important to note that sometimes the textual data collected covered themes not directly expressed in the women’s drawings. This occurred because each time participants presented their drawings, long group discussions would occur where multiple views were expressed, and these often went far beyond the specific aspect depicted in the drawing. In total, we identified 41 themes that were grouped into eight distinct higher-order themes. Six of these concerned the women’s wellbeing and relationship with others, and the other two their fertility healthcare views and experiences. The themes are listed in Table 1. It is beyond the scope of this paper to provide an exhaustive narrative of these themes. Instead, in an attempt to illustrate how the DrawingOut method facilitated the sharing of personal views and experiences, we will focus on describing some of the ways participants engaged with the activities listed above and how this framed the textual data collected. In some cases, we provide examples of the drawings made and also quotes from the participants to illustrate how they presented their drawings to the group or how these triggered different reflections.

**Table 1:** Themes identified in the thematic analysis.

**Emotional burden of infertility**	**Relational burden of infertility**	**Social burden of infertility**
• Fear, confusion, anger • Uncertainty about the future • Sadness, loss of hope, poor mental-health • Low self-esteem • Double pressure’: personal suffering exacerbated by social burden of infertility	• Men less affected than women • Men can be dismissive of women’s suffering • Infertility hinders *vs*. strengthens the partnership • Social pressure affects the partnership • Support from partner is very important	• Social interactions are difficult and stressful • Childlessness creates a rift from social world • Childless women self-isolate • Men are advised to leave childless/infertile women • People should be sensitive regarding childlessness
**The community**	**Research questions**: **How does infertility affect the wellbeing of women and their relationships with their partner, family and community?** **What are women’s views and experiences regarding fertility healthcare?**	**Views and concerns about infertility**
• Traditional views of women and parenthood • High pressure for parenthood • Women blamed for childlessness and infertility • Boys preferred over girls • Male infertility is taboo		• Infertility is a personal journey • Fulfillment is possible without children • Reservations about adoption as alternative route to parenthood • Desire to know about biological causes of infertility • Myths and misconceptions were common
**Coping strategies**	**Healthcare experiences**	**Support needed**
• Taking comfort in religion • ‘Thinking positively’ • Being persistent in trying to get pregnant • Caring for oneself • Being busy and active • Focusing on other life goals	• No perceived discrimination • Lack of interpersonal skills • Lack of empathy towards socio-cultural issues • Good care experiences happen • Pressure/demands on NHS	• Education, fertility education and awareness • Engagement with religious leaders • Counseling • Training in socio-cultural issues and Training in interpersonal skills (healthcare professionals) • Sexual education

Readers interested in an exhaustive description of the themes presented in this table should email the corresponding author.

#### Discussion of infertility related images

All women were able to identify at least one image with which they connected emotionally or conceptually. Several of the women identified with a comic that represented the strained social interactions resulting from infertility by means of a rift metaphor. Indeed, some women replicated this metaphor later on in the workshop, to talk about their relationship with their partner, family or community. Talking about this image, the women stated that they experienced high pressure to have children from their communities. They considered that this pressure was the result of traditionalist views of family and parenthood, whereby children are highly valued and women perceived almost exclusively as caretakers. The women commented that other members of their community, particularly the older generation, were often confrontational and made many insensitive comments. The women also discussed how they coped with such comments.

- *Yeah, I had situations like these because I don’t have children… It has been a very difficult question for me everywhere I go… you know it’s like you get to meet with other ladies and they start talking about their children…*

- *There’s a culture of being interrogated and not knowing the boundaries of personal privacy issues*. *And that’s higher in the BME community rather than in the Britain community*.

Another image chosen by many women depicted a healthcare interaction. This image triggered a long discussion about fertility care that included the narration of multiple personal care experiences. Overall, the women agreed that they were not the object of descrimination within the NHS and that they had experienced a lot of good care, although some also reported being ignored or misunderstood by individual care professionals, who, for example, showed a lack of appreciation of the women’s desire to have more than one child or their reservations about using donated sperm. The participants also commented on a perceived lack of interpersonal skills, particularly among younger doctors.

- *Because I had a daughter*, *a baby girl before that*, *and she was*, *she was about eight years old at that time*, *I felt that they [health professionals] kind of looked down at me*? *You know*. *Why are you even thinking of another one*?

Some women focused on images that depicted the female reproductive system as a way to express a desire to know more about the biological causes of infertility and to ask questions on the topic. One participant said:

- *This [comic] appeals to me because what I am thinking is that […] the person who drew this image is that maybe she had problems with her fallopian tube*, *the problem I have*? *So I have a really keen interest on it […] and I am a bit curious about it*, *what could be done to flow them out to get clearer and to open the fertilization*.

#### Self-portrait exercise

This exercise prompted some women to express their inner dialogue about infertility via the use of thought or speech balloons. For instance, some expressed concerns about fertility treatment, focusing in particular on its low success rates and high costs. Many women talked about the ways they coped with infertility. One of the most prevalent coping strategies involved taking comfort in prayer and in an acceptance of God’s will. In this context women drew several objects symbolising their faith, such as a mosque, the Quran, candles or praying mats. One woman, explainign her drawing said:

- *[Infertility] it’s like you’re behind a door*, *behind a wall and you know the world is outside*, *you just feel really alone but*, *ah*, *you know with my faith I feel*, *you know*, *I’m able to turn to God and to ask for good things*.

Other coping strategies included taking good care of one’s physical appearance, both as a form of self-care and as a way of not revealing distress and suffering to others, and keeping busy and focusing on other life goals. These women’s self-portraits typically showed them wearing earrings or high heels that were an explicit reference to this element of self-care.

#### Visual metaphor drawing exercises

**Fig 1:**
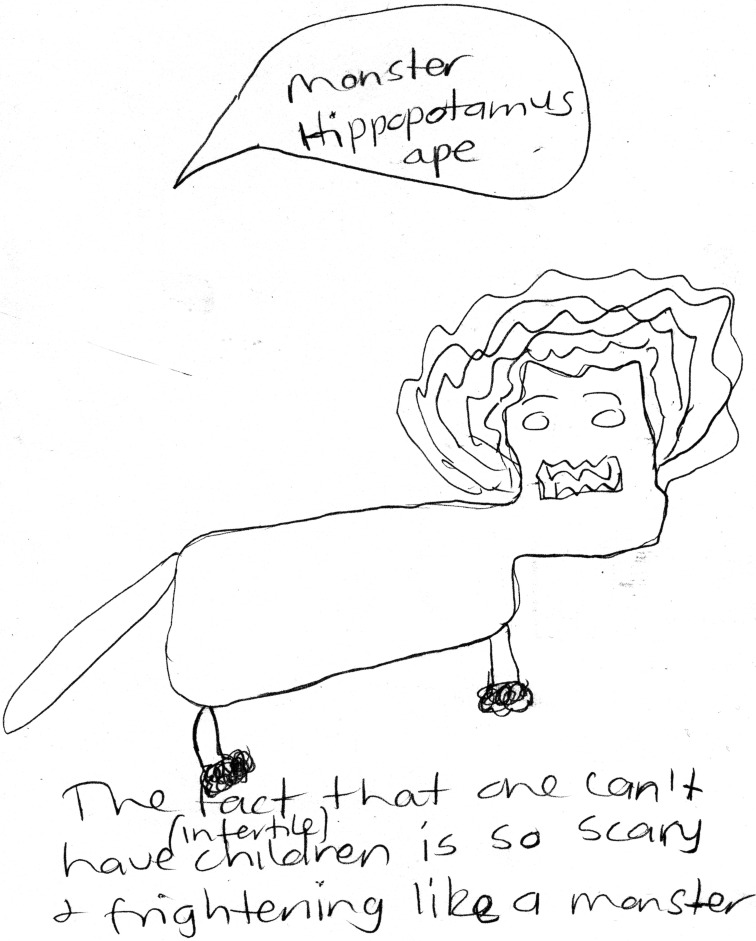
Drawing ‘monster hippopotamus ape’.

These exercises allowed women to express a range of negative emotions caused by their infertility. They tended to use scary animals (e.g., snakes, spiders, leeches) to express feeling frightened, confused or overwhelmed. One participant explained:

- *I have put like a monster hippopotamus ape*, *because the moment you are told*, *the moment you are infertile you become so scared*, *it’s something scary*, *something that can cause you to be depressed or to have poor mental health*. (See Fig 1)

One woman drew infertility as a shadow monster to call attention to the need for better fertility education in her community. This theme was then reiterated across the whole workshop. Women saw education as a way of achieving greater autonomy and gender equality, overcoming traditionalist views of women and parenthood, and increasing awareness about the impact of infertility, in order to encourage men and the community in general to be more supportive. They referred specifically to the need to increase awareness of male infertility and to make men more willing to seek treatment.

- *Infertility is always there*, *wherever you go*, *like a shadow*. *If we educate people the shadow will still be there but much smaller*. *We can be bigger than our fears*.

- *Change the way you up bringing girls separately from boys*, *it’s not something that you can change when you are thirty […]*, *when you’ve been brought up in a culture and an idea that the sole purpose of your existence is to get married […]*, *you know*.

The women also drew different weather manifestations to represent their emotions, with gloomy weather generally representing sadness, anger, loss of hope and uncertainty about the future, and sunny and warm weather representing happiness and fulfilment. Some women, however, used rain as a symbol of fertility, as in their home country rain ensures their agricultural land fertility and is thus a symbol of hope.

#### Free drawing session

Women opted to do different things during the free drawing session. Some just repeated a previous composition in bigger scale and better quality. Others integrated different elements from their previous drawings to make a more complex narrative of their experience. Others came up with a new drawing composition focusing on an aspect that they had not yet drawn about.

**Fig 2:**
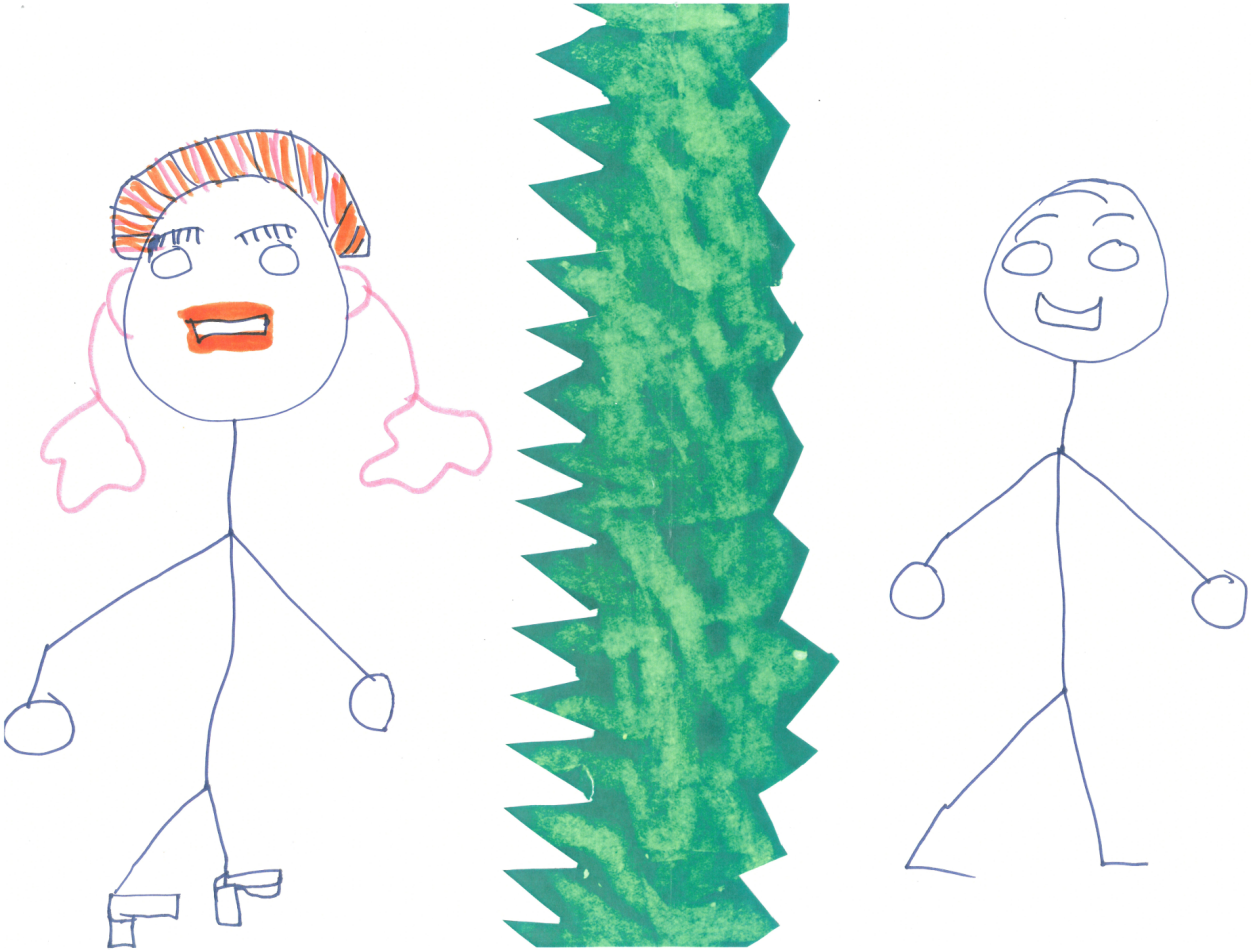
Drawing containing rift metaphor.

As stated above, some women replicated the rift metaphor, or they used similar images, such as a valley, a wall or prison, that is, to show the separation of affected women or couples from the ‘fertile world’ (Fig 2). Commenting on her drawing of infertility as a rift valley, one participant described her deeply painful sense of social isolation:

- *This is the community […] and there’s like family*, *friends*, *schoolmates*, *in-laws*. *So this is me here*, *[…] there is this unconscious divide between*, *this rift valley existing in terms of the stereotypes that they think about you*, *the stigma that they think you have*, *a problem*. *And in the concept*, *it’s that they expect that it should be why your self-esteem*, *but when they see you still confident they think you really don’t care*, *you should be the problem*, *and they think you should always be weighted down*, *so when you are proud*. *So in a way it*, *it*, *it affects your love because when they influence your husband to be against you somehow*, *it might not be obvious*, *but somehow there is an indirect influence*, *especially from the in-laws*.

**Fig 3:**
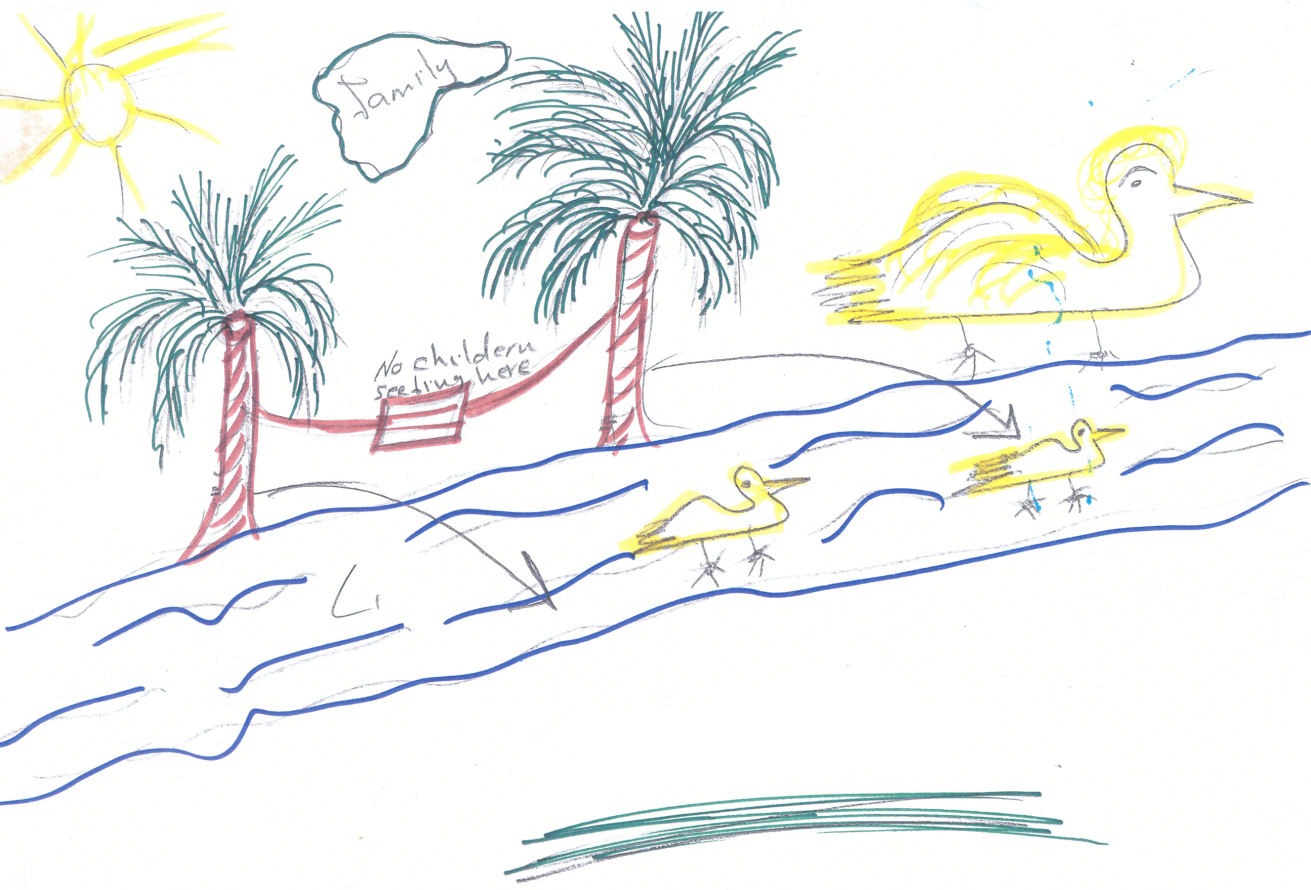
Drawing containing journey metaphor.

Several participants used a journey metaphor to describe their infertility experience: At the start there is the hope of becoming a mother, and difficulties conceiving are imagined as impediments on a long, arduous journey. At the end of the journey there is either the longed-for child, or, in some cases, a sense of having achieved contentment by focusing on other goals. Most participants expressed the belief that women could be fulfilled in life even without having their own children, particularly through education and a professional career. As examples, one woman drew her experience of infertility in terms of climbing up a mountain, while others represented themselves as ducks swimming on a river (Fig 3), or birds flying into the sky. The common element of these drawings was a sense of hope in the face of change or adversity.

Regardless of the main focus of the drawings, many included cultural and religious symbols. While cultural symbols such as the hijab and words written in the participants native language were used to reinforce self-identity in the drawings, religious symbols such as the Quran and praying mats were also used to describe the great sense of comfort participants derived from their religious faith. Another commonality was that many drawings also included the participants’ husbands. Such drawings tended to focus on how infertility affected both members of the couple and/or their relationship.

### Dissemination

We worked with the artist Paula Knight to produce a booklet to disseminate the research findings. This A5 16-pages booklet is structured to reflect the eight higher-order themes identified in the workshop discussions, and it uses participants’ drawings and words (i.e., representative quotes of the lower-order themes) to convey their views of infertility. It also provides a brief general description of the participants and a toolkit with infertility-related information and support for readers. The artist worked on the graphic design of the booklet and introduced minor changes to participants’ artwork (e.g., adding colour) in order to increase the booklet’s visual coherence and attractiveness. The booklet was presented to the workshop participants, and we incorporated a few minor changes suggested (adding colour to one image, increase font size) before its final production and dissemination to various stakeholders and members of the public, including in the women’s own communities. The booklet is available in English and Welsh, both in a paper format and as a digital file for downloading (see S1 File).

### DrawingOut evaluation

A two-hour follow up session was conducted with six of the workshop participants. We asked the women several questions: ‘What was your favourite part of the workshop and why?’; ‘What did you not enjoy so much in the workshop and why?’; ‘How did you find the drawing activities?’; ‘Have you had any experience of drawing before the workshop?’; and ‘What was the most interesting or surprising thing for you in the workshop?’. Most women stated that their favourite part of the workshop was the drawing: it allowed them to convey their views and feelings more efficiently and effectively than if they had been obliged to express themselves just orally; they believed that drawings had a more immediate effect on people; and it made it easier and more enjoyable to talk about such a difficult topic. All women said they had enjoyed the drawing activities, regardless of their previous drawing experience. The participants valued the opportunity to share their own experiences and hear about other women’s experiences of infertility. They also appreciated that the workshop did not only focus on negative aspects of their experiences, which they found empowering. Some of the women worried that members of their community might find out that they had attended this workshop and that this might lead to gossip. They thought that similar anxieties were likely to have kept other women from participating. One woman suggested that men should have been invited to participate too, as this might have encouraged better communication within couples, but others disagreed, stating that they would not have felt comfortable speaking about such intimate issues in the presence of men. When questioned about what was surprising or interesting, some women mentioned information about the biological aspects of infertility. They proposed that a gynaecologist might be invited to attend any future workshops. One participant was surprised by the plurality of interpretations that were generated by the comic strips (activity 1). Two other participants said they felt empowered by the realization that their experiences were shared by other women in a similar situation.

## Discussion

This article presents the first evidence that DrawingOut can provide an effective means to investigate personal views and experiences about sensitive health topics. The DrawingOut method proved successful in engaging a group of women from diverse cultural and religious backgrounds and with varying levels of language proficiency, eliciting a diversity of views and experiences regarding a sensitive health topic, and creating an enjoyable experience for participants. The method’s efficacy is further demonstrated in the amount and richness of the visual data that was produced and shared with different audiences in the form of an engaging booklet. These findings suggest that DrawingOut can constitute a new approach to qualitative and mixed research that may be able to overcome some of the limitations of more traditional methods.

The DrawingOut method was successful in engaging a minority group with varied levels of language proficiency. It should be noted that many participants were from a Muslim background, which was raised in our research development as a potential constraint on using a drawing method, because some Muslims’ beliefs prohibit representations of people (human figures) [30]. We discussed this in the workshop, and some participants noted that they managed this individually, by drawing objects instead of people, for example. Despite these specific cultural constraints, all participants engaged well with all activities, as evidenced by the rich discussions recorded and the amount of drawings created. Participants themselves validated the method, as they stated in the evaluation session that they had enjoyed the workshop, that they found the drawing component very appealing, and that it had made it easier for them to talk about a topic which they would otherwise have found distressing.

In line with the expressed value of drawing as a projective technique [17], our pilot study also supports the claim that DrawingOut is an effective method to research sensitive health topics. The use of the method resulted in the production of a large set of themes that covered a wide range of views and experiences. Some themes emerged when participants were asked to comment on infertility-related comic strips (e.g., the impact of the community), others emerged due to the exercises that prompted participants to draw their own visual metaphors (e.g., emotional burden of infertility), and others in the discussion that followed participants’ presentation of their artwork. Indeed, these presentations tended to trigger extended discussions where multiple themes emerged, many of which were not explicitly represented in the artwork. It was also interesting to see that some of the drawings were used to communicate unmet needs around infertility, such as the need for more information about the biological causes of infertility. This suggests that participants were able to put their own agendas forward, at least to some extent.

Only a comparative study would have allowed us to ascertain whether the DrawingOut method was able to produce more or richer data compared to other techniques such as interviews or focus groups. Yet, although we cannot equate participants’ engagement with higher quantity or more in-depth disclosure or reflection, it is fair to say that without engagement disclosure will not happen. During the workshop, all the participants engaged in the discussions, in both the smaller and larger group formats, commenting on other people’s views and offering their own. They also provided detailed explanations of their drawings and commented on others’ artwork. In addition, participants made extended use of metaphors in their drawings, which enabled them to share life experiences that might have been hard to put into words. Finally, participants themselves felt that the drawings made it easy to convey their feelings and emotions in a striking manner. Overall, as hypothesized, these data suggest that the DrawingOut method facilitated meaning making [9], not only at the individual level but also at group level, as discussion allowed both for consensual group views to emerge and for more individual or idiocyncratic experiences to be expressed.

In addition, DrawingOut made explicit some of the intersections that exist between the participants’ cultural backgrounds and their infertility experiences. As expected, we found that participants’ metaphors were shaped by their cultural background [14]. More specifically, the women’s drawings included elements with specific cultural meanings and connotations, which were used precisely because of these meanings. For example, how women used rain to express their emotions varied according to their cultural and religious background. While some followed the western practice of using rain to express negative emotions, others drew rain as a metaphor for blessing and fertility, and, while laughing at the irony involved in translating this to rainy Wales, traced the metaphor’s cultural roots to the arid climates of North Africa and South Asia. Drawings of birds, moons, and a tree losing its leaves connected to Urdu poetry; a drawing of a ‘monster hippopotamus ape’ for infertility employed the hippo’s lethal reputation in sub-Saharan Africa; and a drawing of the Quran and a prayer mat represented the comfort one of the participants found in prayer and the acceptance of God’s will. Considering that both words and images may be used to stand for feelings and abstract concepts, our study confirmed that drawings are just as permeable as speech to cultural meanings, and that they can thus serve as a gateway to a better understanding of culturally specific interpretations of disease.

Finally, one obvious advantage of our method was in sharing research findings in an accessible and appealing way [18]. Instead of only using quotes from participants in the booklet, we were able to combine these with drawings to encourage a more empathic engagement of readers with the research findings. Another advantage resulting from the use of the participants’ artwork is that the graphic elements and visual metaphors of the booklet are representative of their socio-cultural background, which might make them more appealing to other members of their minority ethnic communities. It is expected that the booklet will facilitate reflection and dialogue around the sensitive topic of infertility [15] beyond the workshop itself. For instance, the launching event of the booklet prompted discussion within the charity Women Connect First about how infertility affects couples and is often not publicly discussed.

Despite the novelty and strengths of the DrawingOut method presented, some limitations need to be acknowledged. First, although DrawingOut allows participants without language proficiency to express their views through drawing, the method implies that they still have to explain their drawings verbally. Due to funding constraints we could not pay for translation in our pilot study. However, an organic translation emerged, where fluent English-speaking participants would translate the explanations offered in their shared native language by those who had no or very little language skills and/or the text they had written on the artwork. Other participants expressed themselves in English despite their limited proficiency. Overall, we need to acknowledge that some data may have been lost in this process and that the DrawingOut method minimises but does not fully overcome language barriers in research. Another limitation of the method is that moderation is important to ensure that the views of all participants are represented, and not only of those with better speaking and drawing skills. Therefore, as with focus groups, and despite the fact that participants still have an initial input via the individual drawings that precede the group sharing, good moderation is essential to ensure the quality and representativeness of the data collected. Finally, our pilot study also had some limitations resulting from the small sample size and its heterogeneity. Consequently, while our results present a nuanced characterisation of some infertility experiences of women with a minority ethnic or religious status in the UK, they lack sufficient depth to adequately represent the full scope of some of the emergent themes (e.g., how religion shapes infertility experiences) and of the different minority groups represented.

Although we did not set out to explore the potential of the DrawingOut method as a support tool, results from our pilot study suggest that it may have also had some therapeutic benefits for the participants via at least three mechanisms:

1. Normalization of experiences and empowerment: In the evaluation session, the participants expressed that they welcomed the opportunity to share their experiences with other women and to hear about their experiences, and that they felt empowered when they realized their experiences were shared by other women in similar situations;
2. Social support and connectedness: Despite many of the participants already knowing each other through community work, they stated that they had never shared their infertility experiences with each other before and that the workshop created an opportunity for social sharing that was otherwise unlikely to have presented itself spontaneously;
3. Education: Participants appreciated the fact that they had the opportunity to access information, for instance, about the biological aspects of infertility. They suggested that the educational component of the workshop could be enhanced by inviting a health professional to participate.

In sum, drawing techniques have been used before to facilitate engagement of different groups with health related topic, for instance younger people [31] or minority groups [32]. Similarly to the DrawingOut method, many of these studies departed from a participatory research paradigm, using drawings as an inclusive technique to empower marginalised groups in setting their own health research agenda, while simultaneously emphasising the idea that participation is not dependent on artistic skills [33]. Previous drawing-based research can broadly be divided into studies that explore drawing as a research method that facilitates knowledge production [6], both at the individual and group level (shared experiences) [34, 35], and studies that use pictures to support health communication [14]. What is innovative about DrawingOut is that it combines these goals (knowledge production and communication) into a fully participatory process that results in a tangible output that is co-produced by researchers and participants. Further, it relies not only on the use of drawing but is theoretically driven by research on visual metaphors [9]. Previous research has highlighted the potential therapeutic benefits of drawing [34], and the feedback from the participants in our study also seems to support this. However, to the best of our knowledge, no research has been conducted to measure such benefits and therefore this should be addressed in future research.

The DrawingOut method is similar to other qualitative and visual methods in terms of preparatory work and training (none of the four facilitators had any formal training or previous experience with leading drawing workshops). Costs can be kept low if inexpensive drawing materials are used, which should not impact on the quality of the data. It is up to the users of the method to decide on the level of production of the dissemination materials and associated costs. The team is currently exploring ways to adapt the DrawingOut, both in terms of its delivery format (group, individual) and the media used (e.g., photography, collage). The method seems suitable for use with other patient populations, in particular those suffering from stigmatised (e.g., HIV, psychosis) or ‘invisible’ diseases (i.e., without any apparent physical symptoms, such as endometriosis or myalgic encephalomyelitis [ME]). The promising results of our study suggests that the metaphor-centred drawing method may also be a useful tool to study other sensitive topics such as forced migration, armed conflict, and political and social injustice. Finally, the analysis presented in this article focused on the textual data generated/recorded during the workshop, while the drawings were only used to illustrate how the different themes emerged. The analysis of the visual data generated with DrawingOut constitutes another promising avenue for future research, as we have demonstrated in another publication [36].

## Conclusions

The DrawingOut method is a one-day metaphor-centred drawing workshop to collect visual and textual data about a particular (sensitive) health-related topic. Findings from our pilot study suggest that the DrawingOut method can be used to engage people to talk about sensitive health topics, while simultaneously providing them with an enjoyable experience. DrawingOut is a promising participatory method to elicit rich visual and textual data that captures a diversity of views and experiences, with the added benefit that these can be brought together to co-produce engaging outputs for dissemination.

## Supporting information

S1 File Thorns and Flowers booklet. (PDF)
