# Supplementary material for: DrawingOut – An innovative drawing workshop method to support the generation and dissemination of research findings
Source: PLoS One. 2018 Sep 20;13(9):e0203197. doi: 10.1371/journal.pone.0203197 (PMC6147407; doi:10.1371/journal.pone.0203197)
Supplement: S1 File — (PDF) [file pone.0203197.s001.pdf]

THORNS and FLOWERS

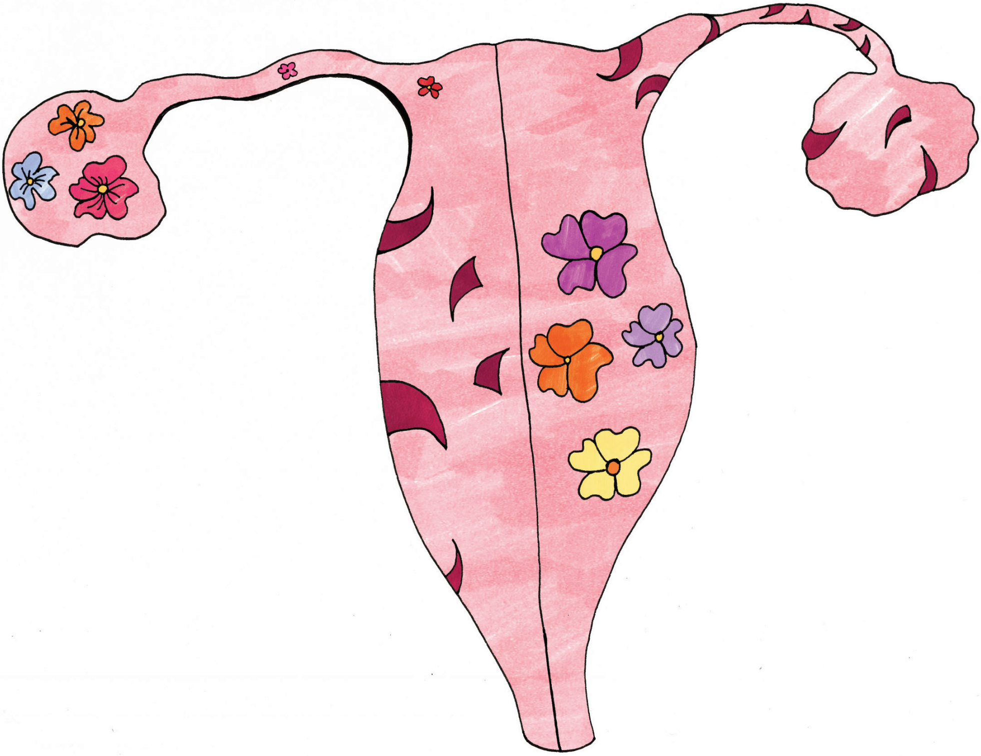

Infertility Experiences of Black and Minority Ethnic Women

This booklet is one of the outputs of a research project that was funded by the Welsh Crucible programme and conducted by a group of female academics at the universities of Cardiff and Aberystwyth.

Research team
Sofia Gameiro, School of Psychology, Cardiff University
Alida Payson, Journalism and Media Studies, Cardiff University
Berit Bliesemann de Guevara, Department of International Politics, Aberystwyth University
Elisabeth El Refaie, English, Communication and Philosophy, Cardiff University

In partnership with
Women Connect First, Charity, Cardiff Wales

Booklet design
Paula Knight, Author, Illustrator and Comics Creator

CARDIFF UNIVERSITY
PRIFYSGOL CAERDYDD

PRIFYSGOL ABERYSTWYTH UNIVERSITY

WelshCrucible CrwsiblCymru

What is this booklet about?

The content

Infertility views and experiences of Black and Minority Ethnic women using their own words and artwork.

The women involved

A group of nine Black and Minority Ethnic women living in Wales. All women had previous or current experiences of fertility problems.

The workshop activities

First we used artwork by artist Paula Knight to prompt discussions and elicit the women's views about infertility. Then we conducted a series of drawing exercises, designed to prompt the women to share their views about infertility: If infertility was a creature or an animal, what would it be? If it was a place, what would it be? If it was weather, what would it be? We finished by asking the women to create a larger-scale and more detailed drawing that brought together their most relevant views and feelings about infertility.

The main conclusions

The women talked about

- the negative emotional impact of not being able to conceive,
- how pressured they feel to have children and the stigma they experience when they are not able to conceive,
- their concerns about what they should do to conceive,
- the support they need from their community, health professionals and policy makers,
- how they manage to remain hopeful.

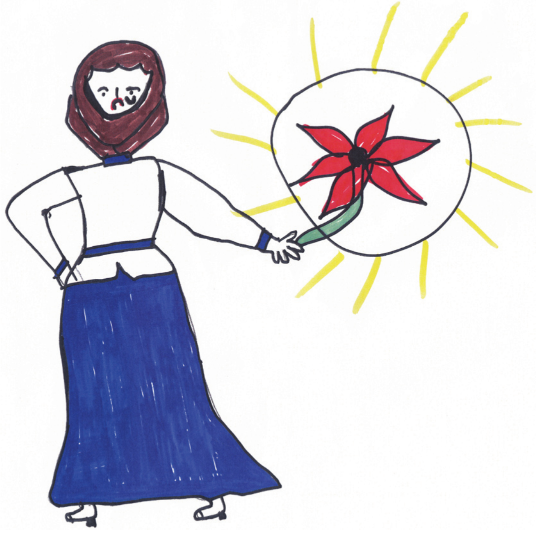

What is infertility like?

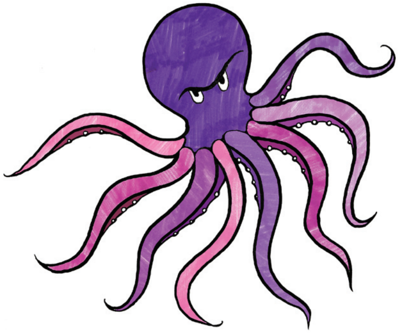

"An octopus, because there are many things going on at the same time and I'm confused."

"Infertility is always there, wherever you go, like a shadow. If we educate people the shadow will still be there but much smaller. We can be bigger than our fears."

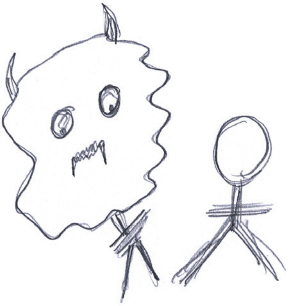

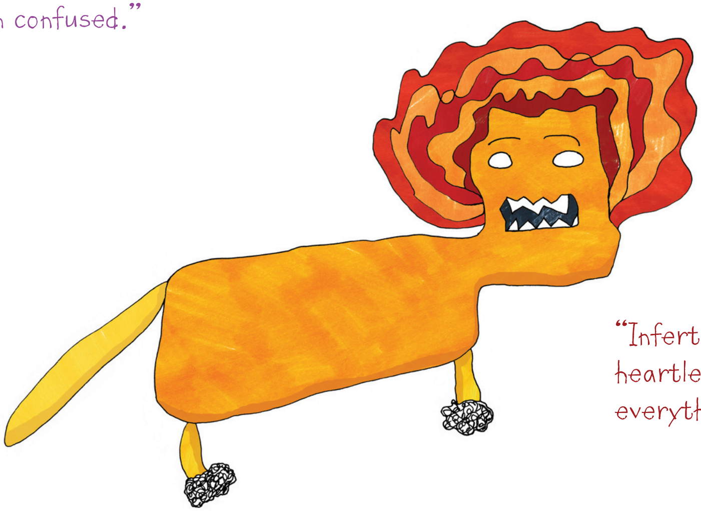

"Infertility is cruel and heartless. It devours everything around it."

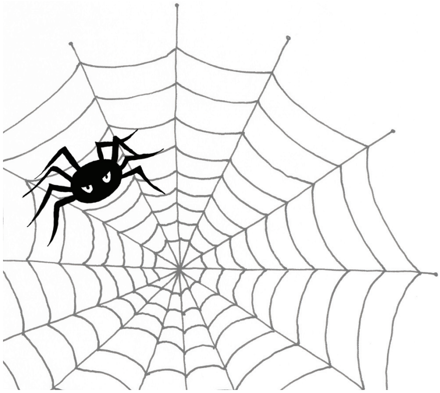

"The moment you're told you're infertile you become so scared... The fear can cause you to have poor mental health."

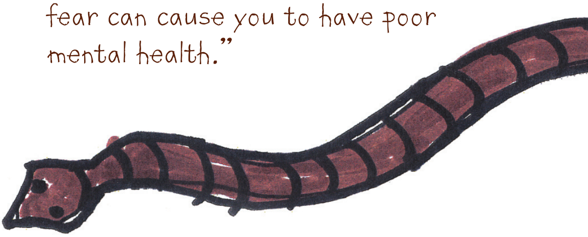

"Nothing grows in the desert."

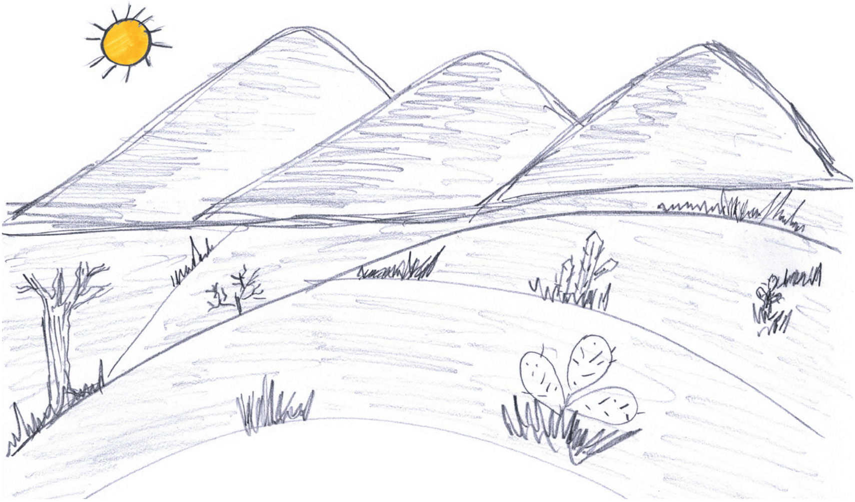

"If you think positively the sun will give you brightness and the power to grow some plants. Even in the desert there can be life."

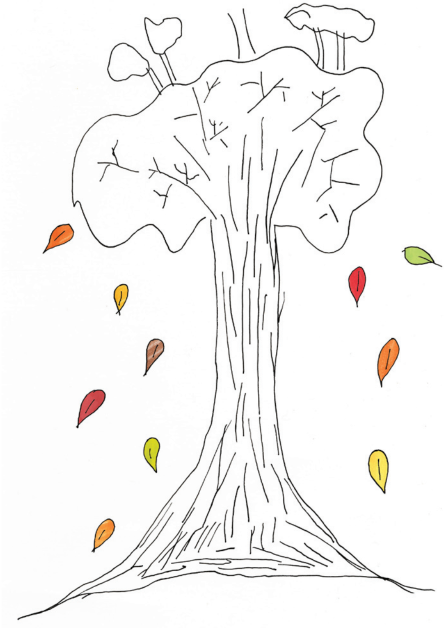

"In Pakistan's Urdu literature infertility is described as the autumn of a woman's body. She can't have children, just like in autumn the trees can't grow flowers and leaves."

"It's always a tug-of-war inside you. It's not just about being pregnant or not. You start thinking you're not suitable for motherhood."

How do others react to my infertility?

"Some older ladies say that now girls don't have children because they want to work and pursue a career. They think girls are making Allah Almighty upset because they're blocking nature's way."

"I think the pressure to have more children is cultural and has nothing to do with religion. When we are pressured by our culture or community we find refuge and escape in religion."

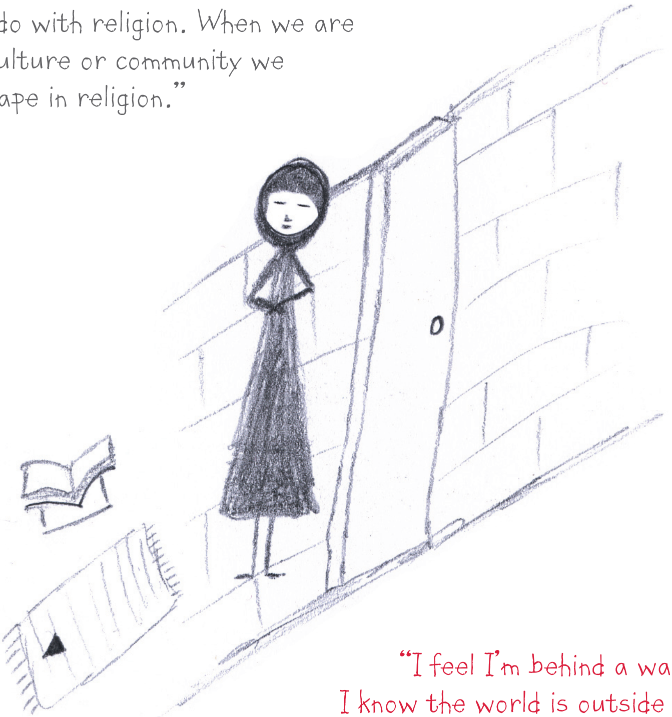

"I feel I'm behind a wall and I know the world is outside but I feel really alone. But with my faith I feel I'm able to ask God for good things."

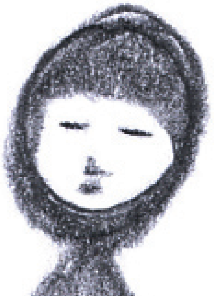

"I may not get support from my mother-in-law or from society, but my husband's support is really important."

"The extended family should support the woman with infertility problems."

"Almost every time I go to a party someone asks if I have children. Maybe they are curious or they don't realize how painful it is or how private it is."

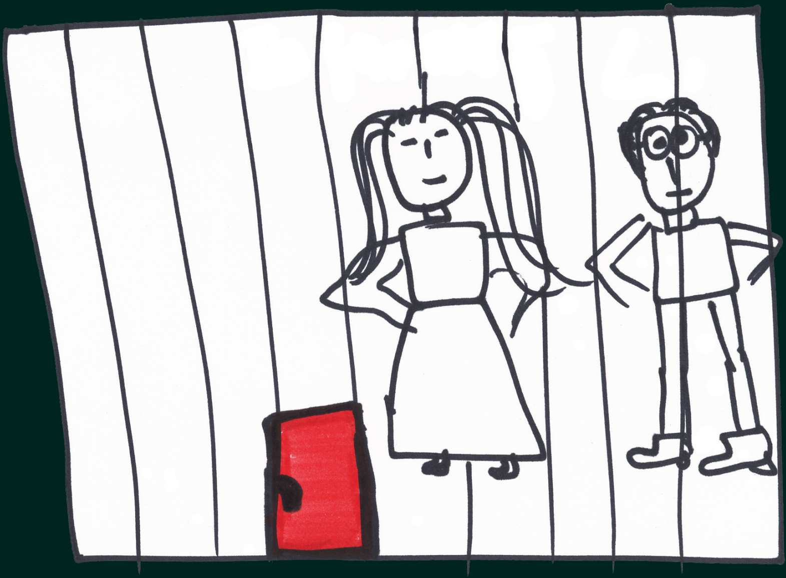

"Infertility is like being trapped in a cage. There's a very small door you can open if you and your husband stay hopeful."

"People treat you as if you have a curse. They think you are the problem, so they start cornering your husband and it affects your love as a couple."

"Change the way you bring up girls separately from boys. Attitudes can't be changed when you are thirty if you've been brought up in a culture where the sole purpose of your existence is to get married."

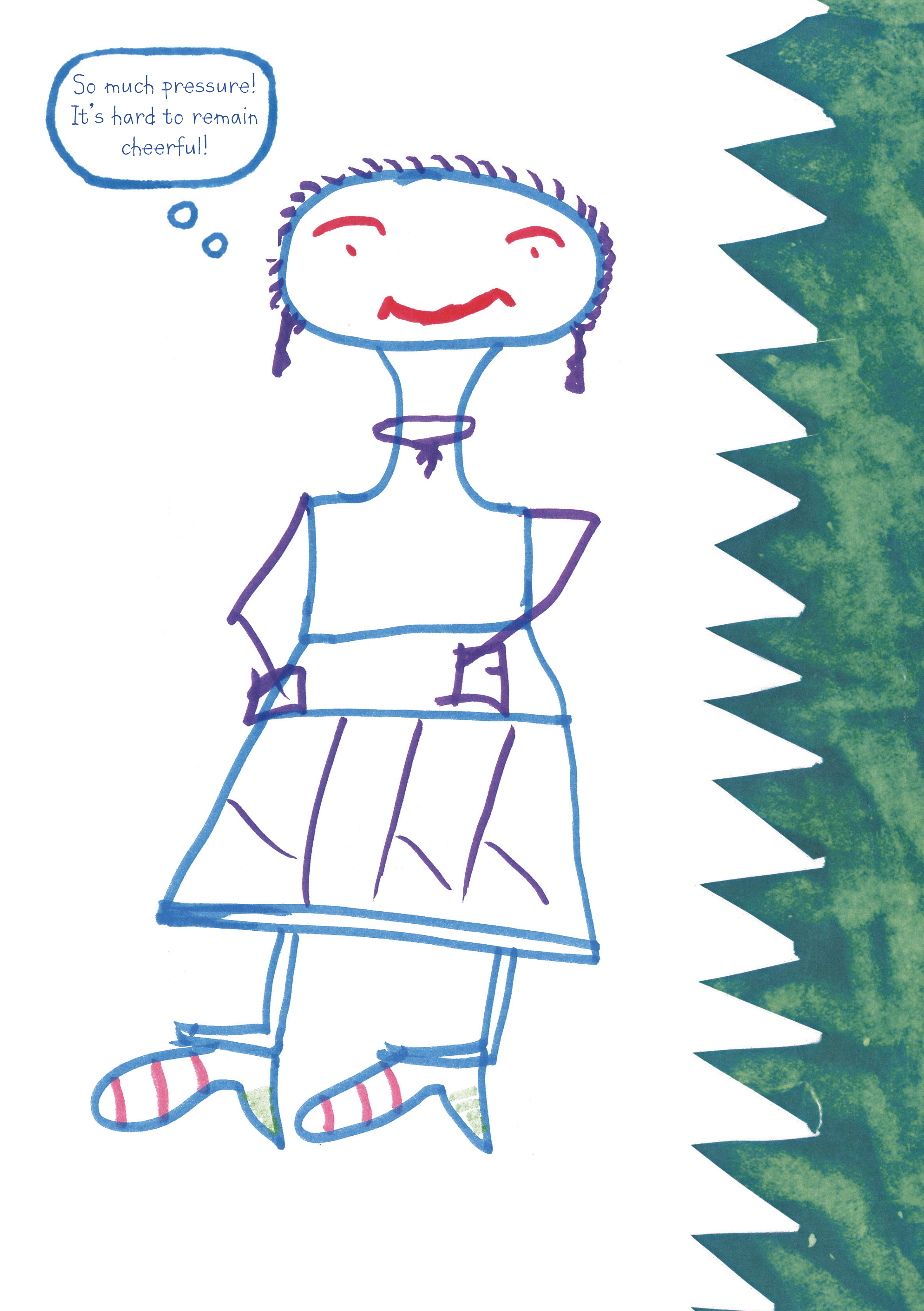
So much pressure! It's hard to remain cheerful!

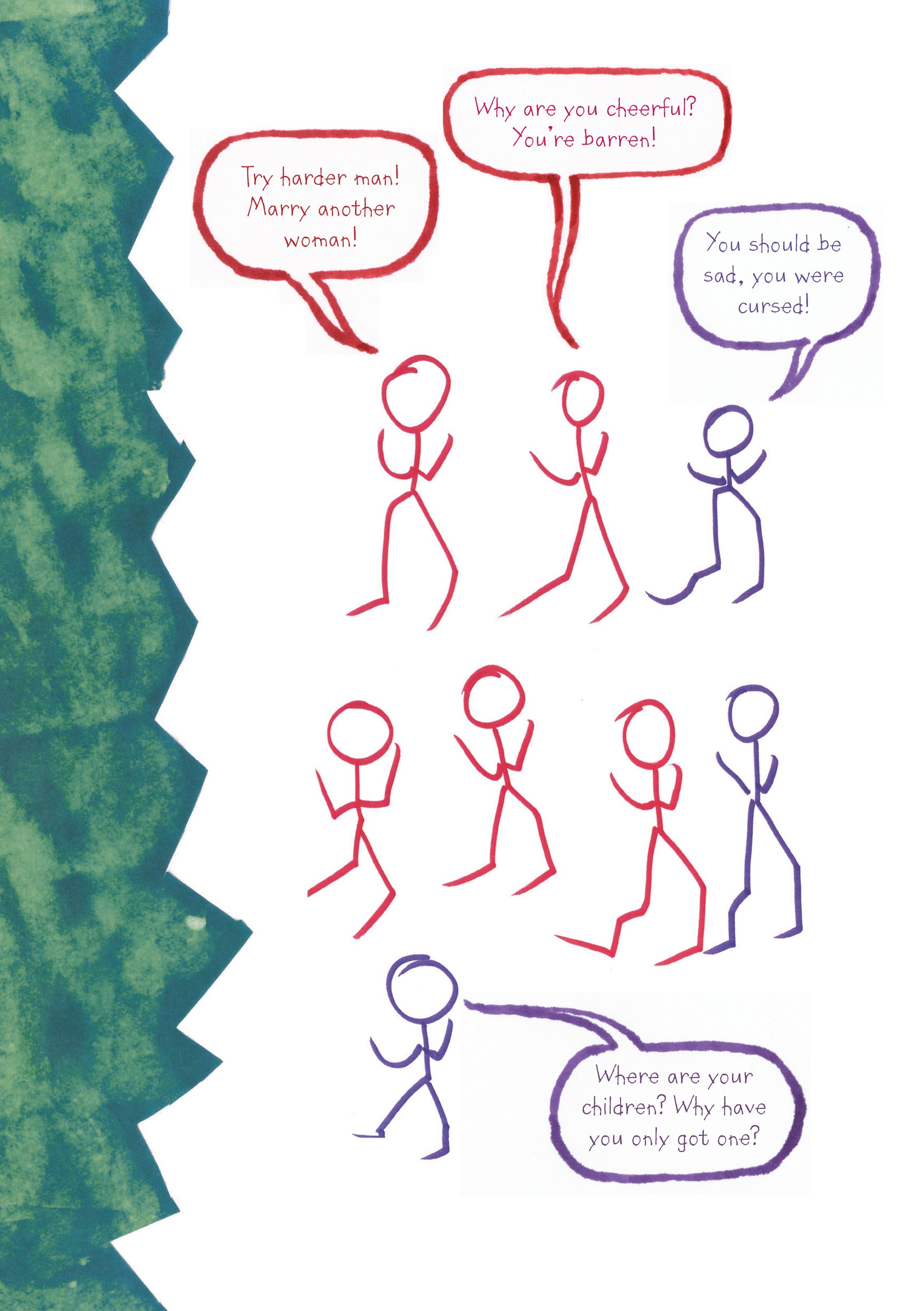
Try harder man! Marry another woman!
Why are you cheerful? You're barren!
You should be sad, you were cursed!
Where are your children? Why have you only got one?

What shall I do to get pregnant?

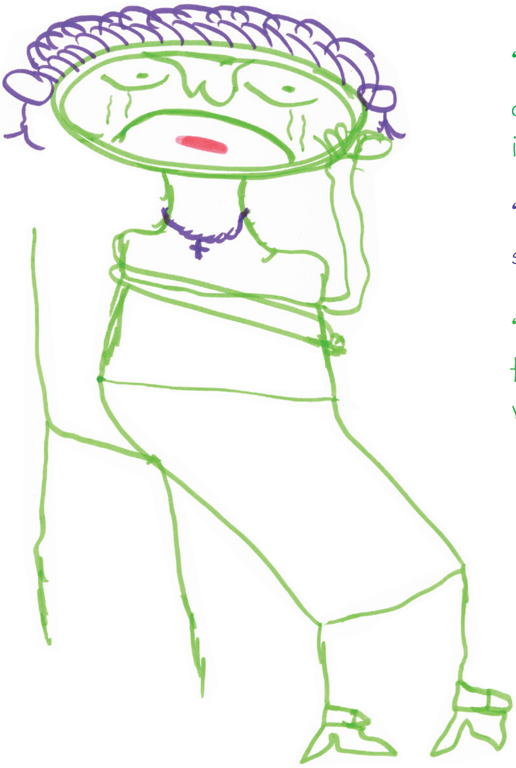

"My friends tell me that until I'm 45 I don't need to worry, but I'm not sure it's true."

"If I forget about it and stop being stressed, might it then just happen?"

"If I trust God and put my faith in Him, if I pray then I am blessed and I will get a child."

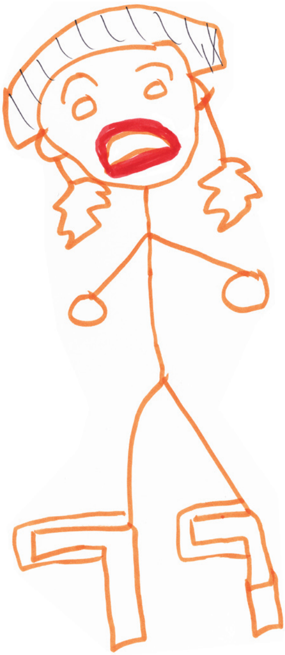

"My hope is to find the right treatment to get pregnant."

"I would like to do treatment, but I have a friend who spent over twenty thousand pounds to have her baby."

"I have a problem with my fallopian tube, I am curious to know what can be done to allow fertilization to happen."

What help do I need?

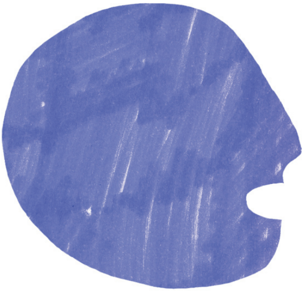

"There is no support for women who end up depressed or with other mental health problems. We need awareness-raising sessions and a support mechanism for these women."

"We need more sex education because a lot of couples, especially from Muslim backgrounds, are sexually inexperienced and so often the infertility is due to their lack of knowledge and skills."

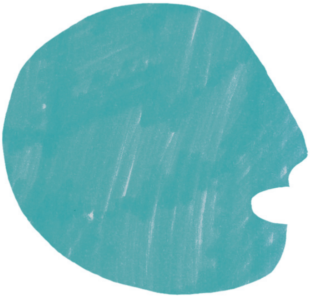

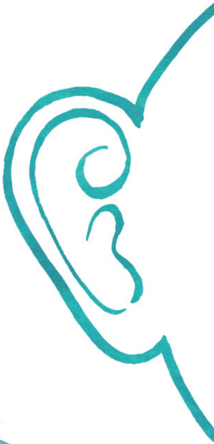

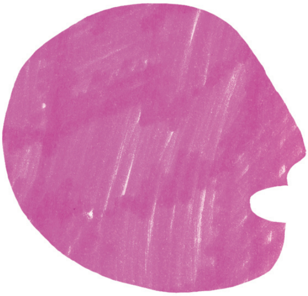

"One advice I'd have for health professionals treating infertile women is to look out for signs of psychological or emotional abuse."

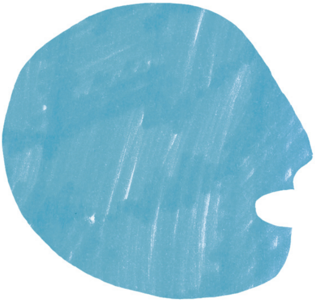

"Health professionals should listen more because at times they miss what the patient is really saying and they give advice that is not appropriate."

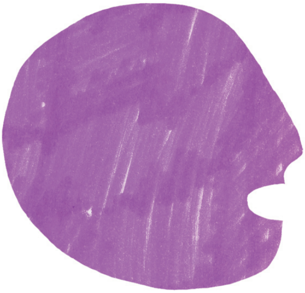

"I think a good suggestion would be for health boards to start engaging with religious places because people, and men in particular, are more receptive at these places."

How can I live with infertility?

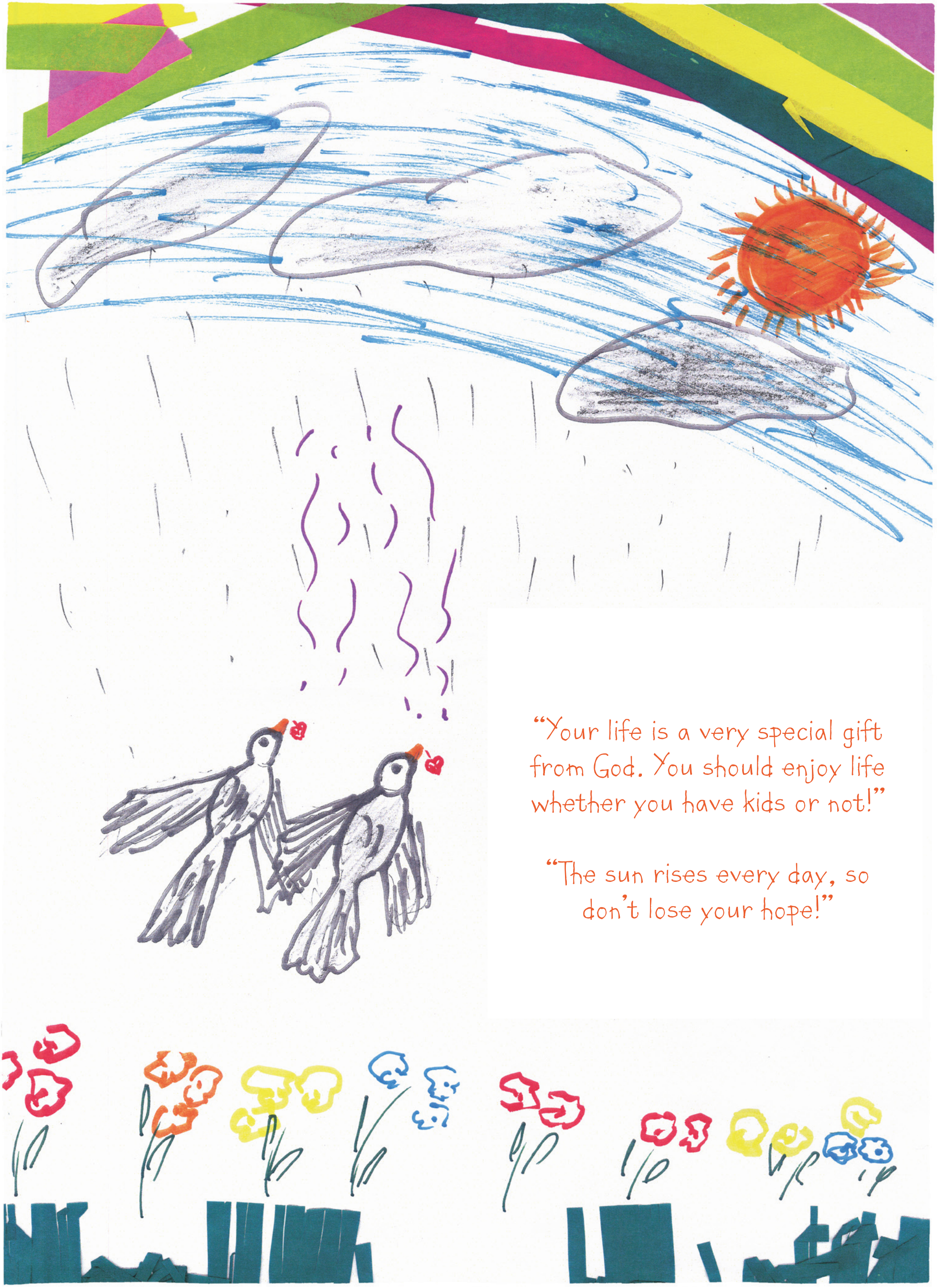

"Your life is a very special gift from God. You should enjoy life whether you have kids or not!"

"The sun rises every day, so don't lose your hope!"

"If you're not pregnant, is that your fate? Is that what is written for you? And maybe you are going against your faith if you try too hard?"

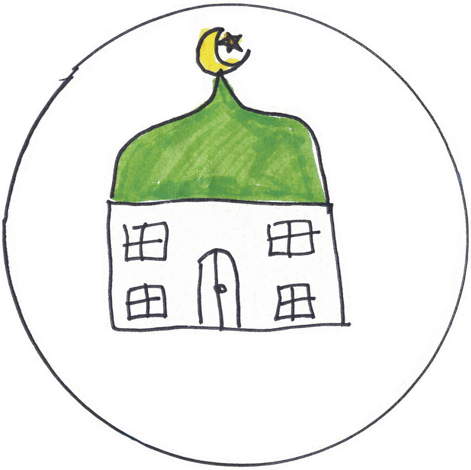

"It helps to be busy. I am volunteering here, volunteering at another charity shop, I just have Sunday for my own house."

"Things have been hard but I still have my high heels on, my necklace and my hair done, you know?"

"I think women can shine through other ways than having children, for instance, educate yourself! There are millions of ways of shining."

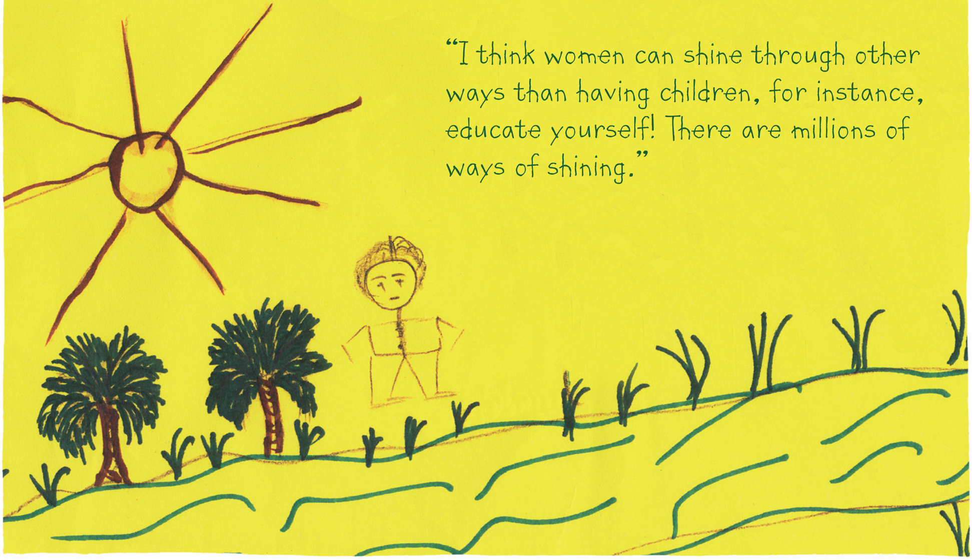

Who created these drawings and spoke these words?

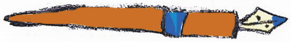

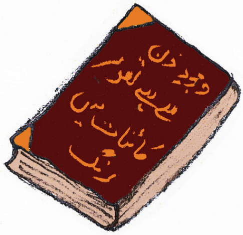

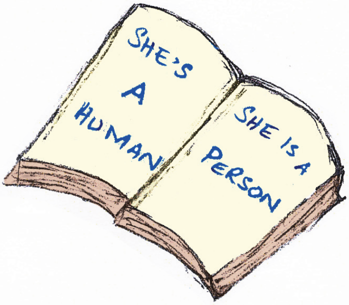

'Women give the universe its colours.'
Dr. M. Iqbal

All of the women who took part describe painful and difficult experiences around infertility. Yet each woman is much more than this experience alone.

Who is she?

She knows the words of a poet by heart. She rides her bicycle beside the river. She volunteers. She reads history, novels, poetry, theology. She collects big earrings. She knows how to coax an eggplant to grow in a cool climate. She has travelled widely. She loves to dance, even in the water. She draws beautiful flowers. She feels at home in Wales. She survives on tea. She helps solve problems in the community. She does calculus the way other people do the washing up. She is a member of a church. She finds sanctuary and peace in the Quran. She has a university degree. She studied anatomy and physiology. She knows the ins and outs of computers. She knows four languages. She can write perfect Urdu. She is learning English. She knows some Welsh. She teaches others. She is devoted to her family. She is a beloved friend. She prefers solitude. She is grateful for blessings. She is political. She tells great jokes. She asks the hard questions. She laughs with her hand over her mouth. She hits the table with her hand to show she's serious. She is unique. Infertility, though thorny, forms only one part of her life.

Would you like more information?

Your fertility
http://www.nhs.uk/Livewell/Fertility/Pages/Fertilityhome.aspx

Your fertility status
http://www.fertistat.com

Your fertility related quality of life
http://www.fertistat.com/fertiqol

How to connect with other people
www.fertilitynetworkuk.com
http://www.fertilityeurope.eu

Infertility, arts and comics
https://paulaknight.wordpress.com/comics-3
http://www.fertilityfest.com
http://www.graphicmedicine.org/comic-reviews/good-eggs-a-memoir

Supporting people with fertility problems
https://britishfertilitysociety.org.uk
https://www.nice.org.uk/guidance/cg156
https://www.eshre.eu
https://www.eshre.eu/Guidelines-and-Legal/Guidelines/Psychosocial-care-guideline.aspx
www.bica.net

Supporting South Asian Communities with fertility problems
http://www.dmu.ac.uk/documents/research-documents/health-and-life-sciences/reproduction-research/endopart/infertilitysouthasiancommunitiesprofessionalresourceleaflet.pdf

The research team is not responsible for the content for these external websites or documents.

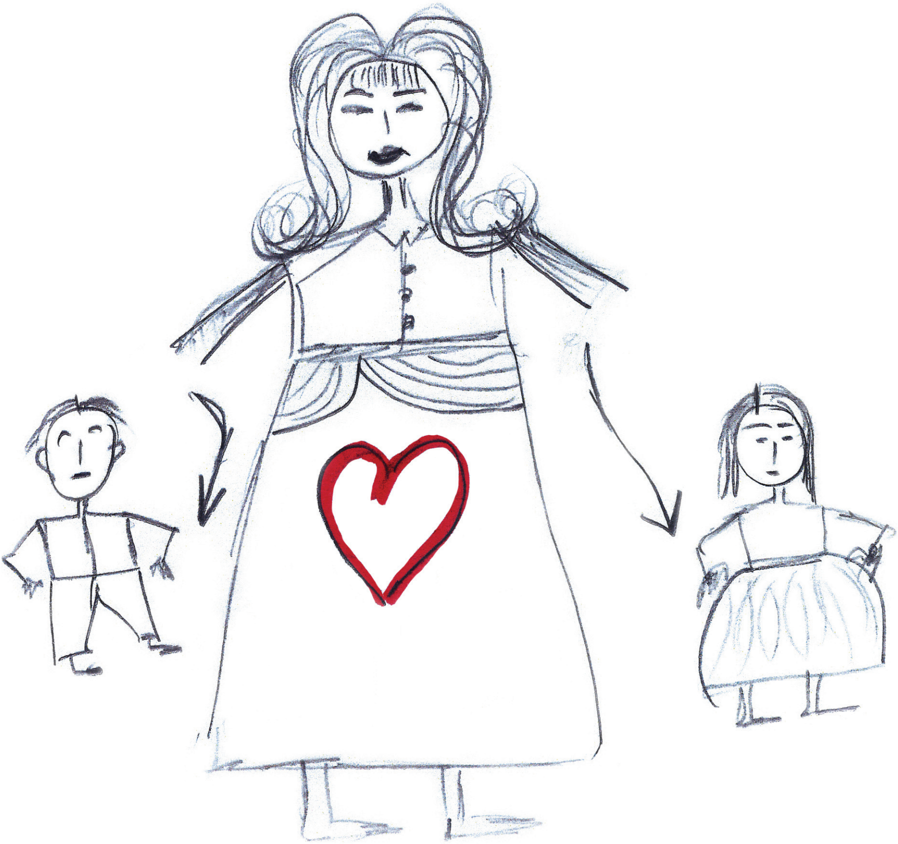

This booklet presents the infertility views and experiences of Black and Minority Ethnic women using their own words and artwork.
